# Neutral and Climate-Driven Adaptive Processes Contribute to Explain Population Variation in Resin Duct Traits in a Mediterranean Pine Species

**DOI:** 10.3389/fpls.2019.01613

**Published:** 2019-12-13

**Authors:** Carla Vázquez-González, Xosé López-Goldar, Rafael Zas, Luis Sampedro

**Affiliations:** Misión Biológica de Galicia, Consejo Superior de Investigaciones Científicas (CSIC), Pontevedra, Spain

**Keywords:** adaptive variation, climate gradients, conifer defences, genetic variation, neutral variation, *Pinus pinaster*, resin canals

## Abstract

Resin ducts are important anatomical defensive traits related to biotic resistance in conifers. Previous studies have reported intraspecific genetic variation in resin duct characteristics. However, little is currently known about the micro-evolutionary patterns and adaptive value of these defensive structures. Here, we quantified inter-population genetic variation in resin duct features and their inducibility in *Pinus pinaster* and assessed whether such variation was associated with climate gradients. To that end, we characterized the resin duct system of 2-year-old saplings from 10 populations across the species’ distribution range. We measured axial resin duct features (density, mean size, and percentage conductive area of resin ducts) and their inducibility in response to methyl jasmonate. Genotyping of single nucleotide polymorphisms allowed to account for the population genetic structure in our models in order to avoid spurious correlations between resin duct characteristics and climate. We found large inter-population variation in resin duct density and conductive area, but not in their inducibility. Our results suggest that population variation in the percentage conductive area of resin ducts likely arise from adaptation to local climate conditions. This study highlights the adaptive relevance of resin ducts and helps to shed light on the micro-evolutionary patterns of resin-based defenses in conifers.

## Introduction

Conifers have developed a broad array of physical and chemical defensive traits and strategies to reduce the damage caused by a large and diverse community of antagonist organisms (Franceschi et al., 2005; Mason et al., 2019; Whitehill et al., 2019). Among them, resin ducts are the structures that produce and exude the oleoresin (Fahn, 1979), an effective terpenoid-based chemical defense against insects and pathogens (Keeling and Bohlmann, 2006). Resin duct characteristics (*e.g*. area or abundance) are considered important proxies of defensive investment in conifer trees because they have been positively associated with enhanced resistance against insects (Kane and Kolb, 2010; Ferrenberg et al., 2014; Hood et al., 2015) and pathogens (Christiansen et al., 1999; Krokene, et al., 2003). Larger resin ducts may enhance resistance because small increases in resin duct lumen area can considerably increase resin flow (Schopmeyer et al., 1954). Accordingly, several studies have reported a positive correlation between resin duct investment and resin flow (Lombardero et al., 2000; Rodriguez-Garcia et al., 2014; Hood and Sala, 2015). Besides their role in oleoresin accumulation, resin ducts act as an anatomical barrier, and their spatial distribution may be crucial in determining the success of an attack. Supporting this idea, Boucher et al. (2001) reported that a denser network of smaller resin ducts was positively correlated with resistance to *Pissodes strobi* Peck because it leaves fewer undefended gaps for the entrance of organisms. In contrast, bigger and fewer resin ducts would lead to larger gaps, favoring insect accessibility to feeding tissues (Reed et al., 1986).

Resin ducts are present constitutively (*i.e.,* in the absence of any biotic stress), and are also induced in response to damage signaling. Several external stimuli have been reported to induce the formation of "traumatic" resin ducts, including insect attacks, pathogen infestation, or wounding alone (Franceschi et al., 2000; Nagy et al., 2000; Chen et al., 2018). Resin ducts can also be induced by the application of methyl jasmonate (MJ) (Franceschi et al., 2002; Martin et al., 2002; Krokene et al., 2008; Moreira et al., 2015b), a hormone implicated in damage signaling and defensive responses (Hudgins and Franceschi, 2004). Several studies have reported increased resistance against insects and pathogens by means of resin duct induction with different elicitors. For instance, pre-inoculations of *Endoconidiophora polonica* (previously *Ceratocystis polonica* (Siemaszko) C. Moreau) or MJ application in *Picea abies* (L.) Karst trees enhanced traumatic resin duct production and biotic resistance to bark beetles and their associated pathogens in later infections (Christiansen et al., 1999; Franceschi et al., 2002; Krokene et al., 2003; Erbilgin et al., 2006; Krokene et al., 2008). The development of traumatic or induced resin ducts implies, however, a cellular differentiation process at the cambium that can take several weeks (Nagy et al., 2000). Thus, induced resin ducts may not fully develop in time to avoid the effects of an initial attack.

Research on tree defenses has become a priority given the rising biotic stress caused by the progressive expansion of native and newly arrived alien antagonist organisms (Raffa et al., 2008; Santini et al., 2013; Early et al., 2016). Moreover, abiotic stress driven by climate change can also affect biotic resistance in tree species, because more limiting conditions can affect resource allocation to defenses (Herms and Mattson, 1992; Sampedro et al., 2011; Moreira et al., 2015b). Phenotypic plasticity is therefore an important mechanism for dealing with increasing environmental stress in long-lived plant species. However, the speed and magnitude of global change impose serious questions about whether phenotypic plasticity alone will be enough to counter the negative effects of global change (Alberto et al., 2013). Adaptation of tree populations, which ultimately depends on intraspecific genetic variation in functional traits, may be the only way to cope with rapid environmental change (Aitken et al., 2008; Kremer et al., 2012; Alberto et al., 2013). While intraspecific genetic variation in conifers has been widely studied for traits related to growth and abiotic resistance (de la Mata et al., 2012; Gaspar et al., 2013; Grivet et al., 2013), much less is known about variation in anatomical defensive traits such as resin ducts.

Some studies have shown that resin duct features in conifers harbor high intraspecific variation between populations (O’Neill et al., 2002; Zas et al., 2015) as well as within-populations (King et al., 2011; Moreira et al., 2012; Moreira et al., 2015b). However little is yet known about the evolutionary origin of such variation or the adaptive value of this anatomical trait. Provenance trials can be useful to better understand the environmental drivers of genetic variation among populations. For instance O’Neill et al. (2002) reported that climate conditions and geographic variables at the origin of *Picea sitchensis* (Bong.) Carr. populations shaped genetic variation in resin duct characteristics. However, genetic variation includes not only adaptive variation resulting from diversifying selection in fitness-related traits, but also neutral variation due to processes such as genetic drift or gene flow (González-Martínez et al., 2006; Holderegger et al., 2006). It is noteworthy that most studies aiming to assess the adaptive value of quantitative traits by testing the existence of environmental gradients have failed to differentiate neutral from adaptive variation. Accounting for the neutral population genetic structure can, however, considerably change the interpretation of clinal patterns (Xia et al., 2018; López‐Goldar et al., 2019).

Here, we explored inter-population variation in constitutive resin duct characteristics (density, mean size, and percentage of conductive area covered by resin ducts) and their inducibility in *Pinus pinaster*. To determine the adaptive origin of such variation, we assessed the extent to which inter-population differentiation was explained by the climate conditions at the origin. To differentiate neutral from potentially adaptive genetic variation, and to avoid spurious correlations between traits and climate variables, we accounted for the population structure derived from neutral molecular markers in our models. To our knowledge, this is the first study that aims to explain inter-population variation in resin duct characteristics as a function of adaptation to local climate conditions. This knowledge will help to better understand the microevolution patterns of anatomical defenses in conifer species and their relevance in adaptive forest management under global change scenarios.

## Materials and Methods

### Study System

**Figure 1:**
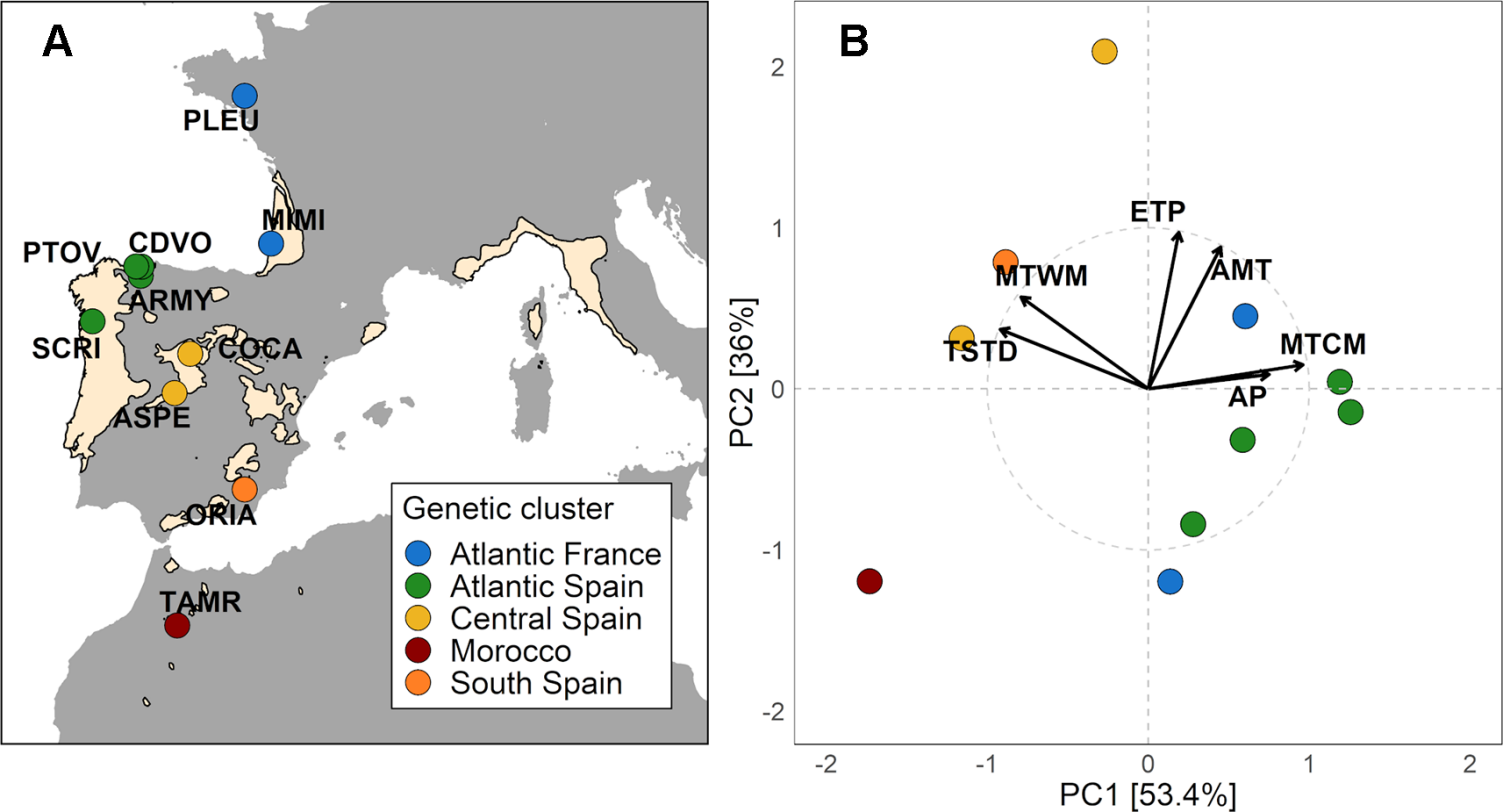
**(A)** Distribution map of *Pinus pinaster* (from EUFORGEN 2009, www.euforgen.org) showing the location of the 10 studied population (See **Table S1**). Colors indicate populations from different genetic clusters based on 1745 SNPs genotyped in 153 randomly sampled and unrelated individuals (from Jaramillo-Correa et al., 2015). **(B)** Principal component analysis of climate variables and plot of the 10 populations in the principal components ordination space. The climate variables included were annual mean temperature (AMT), maximum temperature of the warmest month (MTWM), minimum temperature of the coldest month (MTCM), temperature seasonality (TS), annual precipitation (AP), and potential evapotranspiration (ETP). Higher values of PC1 were indicative of Atlantic climates with higher precipitation and lower thermic oscillation, while lower values were related to Mediterranean climates. PC2 was positively associated with annual mean temperature (AMT) and potential evapotranspiration (ETP).

Maritime pine (*Pinus pinaster* Ait.) is a Mediterranean conifer species of high economic and ecological value in Southwest Europe and North Africa, inhabiting a wide range of heterogeneous environments, from wet-coastal to regions with prolonged summer drought (**Figure 1A**). *P. pinaster* populations are strongly differentiated for traits related to growth, abiotic stress tolerance, reproduction, and chemical defenses (Tapias et al., 2004; Arrabal et al., 2005; Correia et al., 2008; Santos-del-Blanco et al., 2012). Moreover, the demographic history of this species, with multiple isolated postglacial refugia as sources of migration routes, has led to high and geographically structured neutral genetic variation among populations (Bucci et al., 2007; Jaramillo-Correa et al., 2015). Resin duct features in this species have shown large intraspecific genetic variation among populations (Zas et al., 2015) and families within populations (Moreira et al., 2015b). However, the evolutionary drivers of such variation remain unexplored.

### Plant Material, Experimental Design, and Sampling

To explore inter-population genetic variation in resin duct characteristics and their inducibility we used 10 populations of *P. pinaster* from CLONAPIN bank 1 collection (Majada et al., 2011). The populations were spread across the natural distribution range of the species, covering 10 latitude degrees from the southern to northern range ends, representing a wide environmental gradient. At each native population, five mother trees were selected and five open-pollinated seeds were collected from each of them. A total of 250 half-sib seeds with known mother and unknown father were grown under a common environment and comprised the ortet collection (10 population × 5 families × 5 genotypes) (López-Goldar et al., 2018). For the current experiment, two genotypes from 2 to 5 families within each of 10 the population were randomly selected and vegetatively propagated by minicuttings as described in Majada et al. (2011) obtaining two clonal copies, one exposed to methyl jasmonate treatment (MJ) and the other used as a control (CT). When cuttings were 2-years old they were relocated to a glasshouse in the MBG-CSIC (Pontevedra, Spain) and distributed following a split-plot design replicated in two blocks with population as the whole plot factor, and the factorial combination of family and MJ as the split factor. A total of N = 130 plants were included in the experiment (10 populations × 2–5 families × 2 blocks × 2 clonally propagated genotypes, each one subjected to either induction or control treatment). Resin duct induction was performed on plants allocated to the MJ treatment and sprayed with a solution of 25 mM of MJ (Sigma-Aldrich #39270-7) in deionized water with 2.5% ethanol (v/v). MJ was sprayed over the foliage to runoff (~10 ml per plant). Control plants were sprayed with the carrier solution in the same way (López-Goldar et al., 2018). Plants allotted to MJ treatment were transferred to a separate chamber for 6 days to avoid indirect induction effects on control plants by volatile emissions. All plants were watered weekly during the experiment and maintained in the same irrigation conditions to avoid any effects of differential water availability among plants.

One month after the induction treatment, all plants were harvested by cutting the stem aboveground. Then, a short section (ca. 2 cm long) of the main stem at mid height was sampled and stored in FAA (5% formaldehyde, 90% ethanol, 5% acetic acid) and then transferred to 75% ethanol (v/v) as previously reported in Moreira et al. (2015b). Thin transversal sections (40–60 µm) were obtained using a rotatory microtome (Leica RD 2235), stained with 0.01% safranin, and left overnight. Transversal sections were cleaned with progressive ethanol solutions (30, 50, 75, and 90%) for dehydration and to remove the excess of safranin. Finally, the stem sections were mounted on microscope slides and preserved in glycerol.

### Resin Duct Measurements

**Figure 2:**
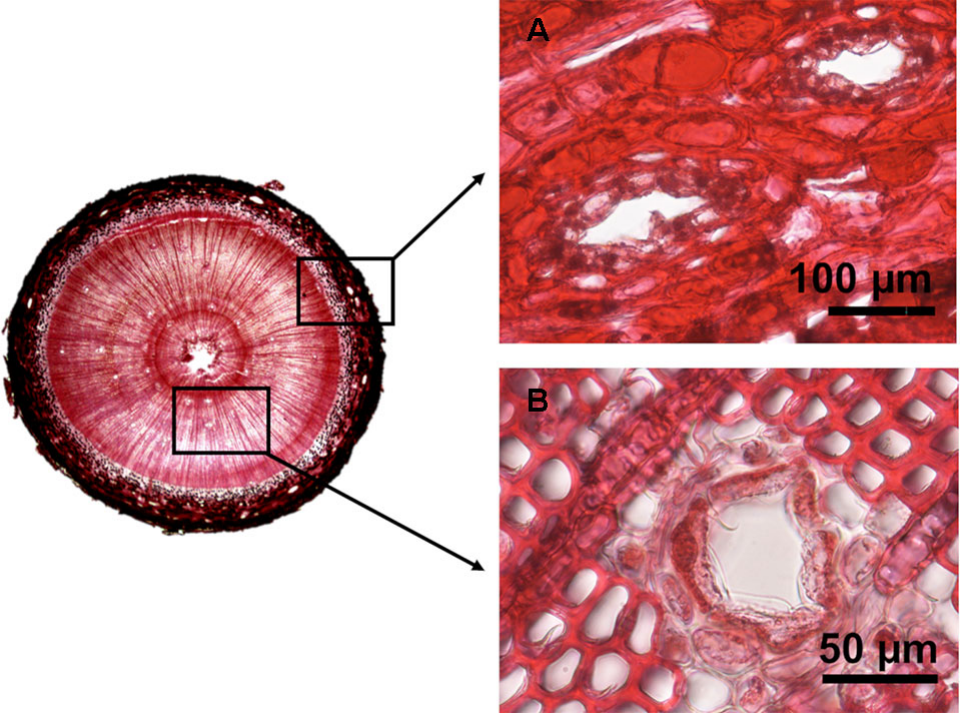
Examples of stem transversal sections of experimental plants showing axial resin ducts in the phloem **(A)** and xylem **(B)** in two year old *Pinus pinaster* saplings.

Images from the transversal sections were obtained with a camera (VisiScope SCM154) coupled to a binocular stereoscope and analyzed using *ImageJ* 1.51k image analysis software (Abràmoff et al., 2004). After calibration with a precision slide (0.01 mm), we measured the area of phloem and xylem and separately counted the number of axial resin ducts in each tissue and measured their lumen area (**Figure 2**). Measurements were taken in two randomly selected quarters of the whole section. In each tissue we calculated the density of resin ducts as the number of resin ducts per square mm of tissue (hereafter RD density – number/mm^2^), the mean area of resin ducts (hereafter RD mean size – mm^2^), and the percentage of the total tissue area occupied by the lumen of resin ducts (hereafter RD conductive area – %) (**Table 1**). Resin duct measurements relative to tissue area represent an estimation of resin duct production in relation to plant growth.

**Table 1:** Description of resin duct characteristics measured in the xylem and the phloem of *Pinus pinaster* plants from 10 populations.

Trait	Units	Description
RD density	RD/mm^2^	Number of resin ducts per unit area in each tissue
RD mean size	mm^2^	Mean resin duct lumen area in each tissue
RD conductive area	%	Percentage of the total tissue area occupied by the lumen of resin ducts

### Statistical Analysis

To have an initial picture of the variation and covariation of the different analyzed resin duct metrics, main descriptive statistics of each variable and Pearson’s correlation coefficient among them were calculated. To explore inter-population genetic variation in resin ducts characteristics (RD density, RD mean size and RD conductive area in the xylem and phloem) and their response to MJ treatment (inducibility), we applied linear mixed models using the *lmer* function from *lme4* package in *R* (Bates et al., 2014). Population (Pop), methyl jasmonate treatment (MJ), and their interaction (Pop × MJ) were included as fixed factors. Family within population (F[Pop]), the interaction between families and MJ (F[Pop] × MJ), and the genotype were included as random factors to reduce the model error rather than to explore genetic variation among families and genotypes. All resin duct variables were log-transformed to meet normality assumptions.

We obtained average climate data for the period 1950–2000 at the origin of populations from regional (Gonzalo, 2007) and global (Worldclim database - Hijmans et al., 2005) climate models. Both models compute gridded monthly climate data with a spatial resolution of 1 km^2^. The model of Gonzalo (2007) was used for Spanish populations due to its higher accuracy modeling climate variables in this region. Climate variables were summarized using principal component analysis as described in López‐Goldar et al. (2019). We used annual mean temperature (AMT), maximum temperature of the warmest month (MTWM), minimum temperature of the coldest month (MTCM), temperature seasonality (TS), annual precipitation (AP), and potential evapotranspiration (ETP). The first principal component (PC1) explained 53.4% of the total variance, with higher values associated with lower thermic oscillation and higher precipitation (Atlantic climates). In contrast, lower values of this index were associated with higher temperature extremes and lower precipitation (Mediterranean climates). The second principal component (PC2) explained 36% of the total variance and was positively correlated with mean annual temperature and potential evapotranspiration (see **Figure 1B**).

To test whether resin duct differentiation among populations was associated with climate, we performed correlation analysis between those resin duct characteristics that showed significant inter-population variation and the climate indices (PC1 and PC2). To that end, population least square means for each resin duct trait were obtained from the afore-mentioned model using the *LSmeans* function from the homonym package in R (Lenth, 2016).

Given the genetic population structure of the species, the non-independence between populations can generate spurious correlations (Desdevises et al., 2003). To account for this source of bias, we retrieved the population structure data (*Q* matrix) based on the genotyping of 126 SNPs from an independent set of clonal replicates (see more details in López-Goldar et al., 2018) publicly available in the Zenodo repository (http://doi.org/10.5281/zenodo.1445313). The *Q* matrix assigns to each genotype a genetic membership coefficient for each of the five genetic clusters (see **Figure 1A**) and was included as a covariate in the afore-mentioned model, as described in Yu et al. (2006). Correlation analyses with climate variables were performed once again, this time using the corrected population least square means for resin duct characteristics, adjusted for the neutral population structure.

## Results

RD density in control and induced plants was higher in the xylem than in the phloem (2.60 ± 0.006 and 0.58 ± 0.002 RD/mm^2^ respectively; mean ± SE., N = 130). However, RD mean size was a 10-fold higher in the phloem than in the xylem (0.01 ± 0.00006 and 0.001 ± 0.000003 mm^2^ respectively; mean ± SE., N = 130). Consequently, RD conductive area was higher in the phloem than in the xylem (0.67 ± 0.003 and 0.25 ± 0.000007% respectively; mean ± SE, N = 130) (see also **Table S2**).

RD density was negatively correlated to RD mean size both in the xylem (*r* = ‑0.41, *p* < 0.001, N = 130) and in the phloem (*r* = ‑0.22, *p* < 0.05, N = 130). Moreover, RD density in the phloem was negatively correlated with RD mean size in the xylem (*r* = ‑0.3, *p* < 0.001, N = 130) and *vice versa*, RD density in the xylem was negatively correlated with RD mean size in the phloem (*r* = ‑0.24, *p* < 0.05, N = 130) (see **Table S3**).

### Inter-Population Variation in Resin Duct Characteristics and Their Inducibility

**Figure 3:**
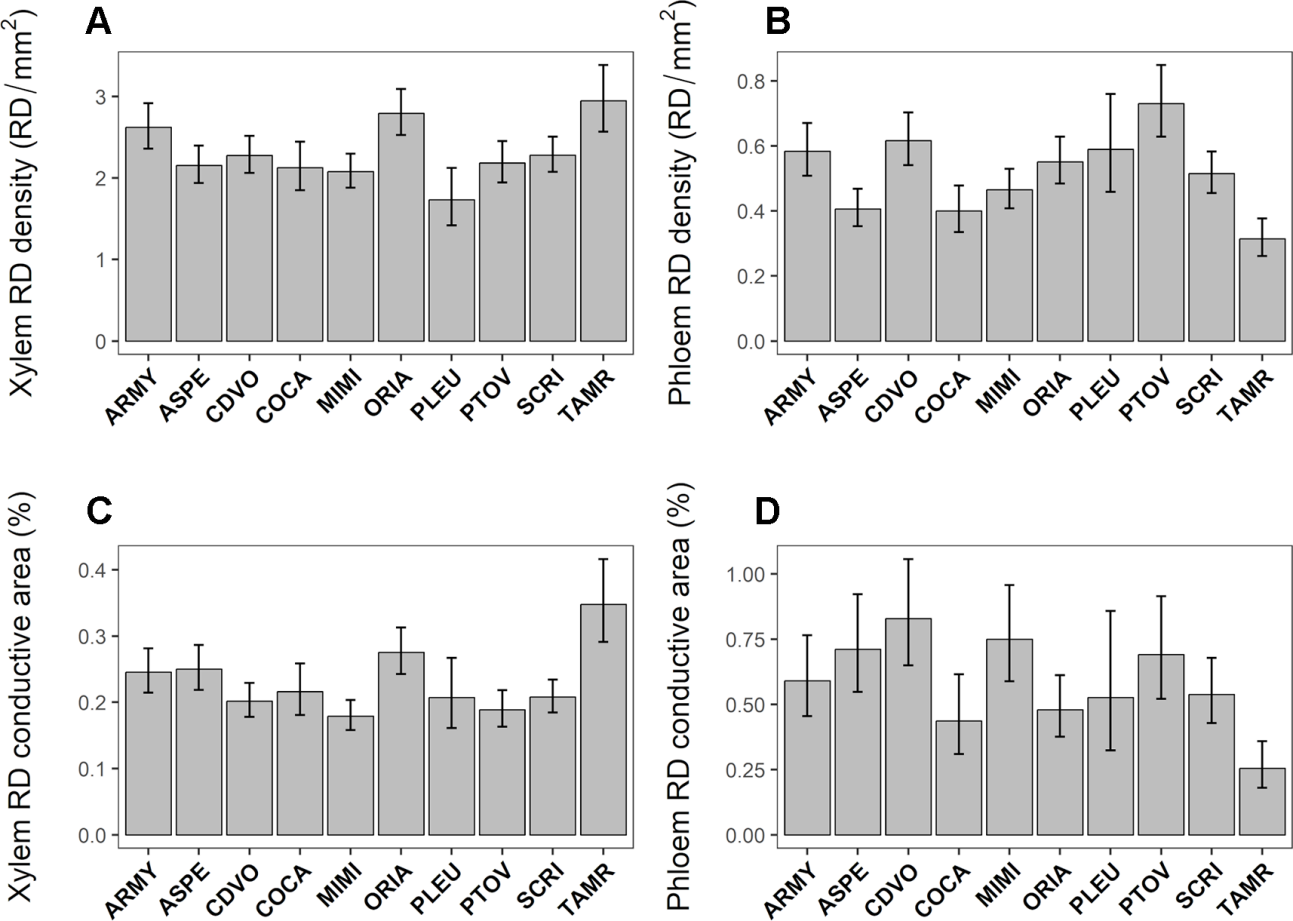
Intraspecific genetic variation in constitutive resin duct density (RD density, RD/mm^2^, panels **A** and **B**) and relative conductive area (RD conductive area, %, panels **C** and **D**) in the xylem **(A**, **C)** and phloem **(B**, **D)** among 10 *Pinus pinaster* populations covering a south–north latitudinal gradient. Bars represent population least square means ± SE extracted from the mixed model (Control plants; N = 63).

RD density in the xylem and in the phloem showed large inter-population genetic variation (**Table 2**). Constitutive RD density in the xylem varied between 1.73 and 2.94 RD/mm^2^ between populations. TAMR was the population with the highest RD density in the xylem whereas PLEU showed the lowest value for this trait (**Figure 3A**). In the phloem, constitutive RD density ranged between 0.31 and 0.73 RD/mm^2^. PTOV and TAMR were the populations that had the highest and lowest value respectively for this trait (**Figure 3B**). Although no significant inter-population variation was found for RD mean size in the xylem and the phloem, RD conductive area was highly variable in both tissues (**Table 2**). Constitutive RD conductive area in the xylem ranged from 0.17 and 0.34 percent between populations, with TAMR and MIMI being the populations with the highest and lowest values respectively (**Figure 3C**). Constitutive RD conductive area in the phloem ranged between 0.25 and 0.82 percent between populations and TAMR showed the lowest value, whereas CDVO had the highest value for this trait (**Figure 3D**).

**Table 2:** Summary of the mixed model testing the effects of Population (Pop), induction by methyl jasmonate (MJ), and their interaction (Pop × MJ) on resin duct density (RD density—RD/mm^2^), mean size (RD mean size—mm^2^) and relative conductive area (RD conductive area—%) in each tissue (xylem and phloem) of *Pinus pinaster* saplings.

	Xylem			Phloem		
	*DF (Num, Den)*	*F*	*p*	*DF (Num, Den)*	*F*	*p*
**RD density**						
Pop	(9, 64.6)	2.5	**0.018**	(9, 65.1)	4.93	**<0.001**
MJ	(1, 53.2)	7.5	**0.009**	(1, 76.1)	2.67	0.107
Pop × MJ	(9, 54.3)	1.9	0.069	(9, 65.1)	0.57	0.814
**RD mean size**						
Pop	(9, 63.9)	1.4	0.227	(9, 59.6)	1.69	0.110
MJ	(1, 55.7)	0.1	0.803	(1, 55.6)	4.81	**0.032**
Pop × MJ	(9, 56.9)	0.9	0.568	(9, 57.0)	0.64	0.757
**RD conductive area**						
Pop	(9, 52.6)	2.7	**0.012**	(9, 59.5)	2.31	**0.027**
MJ	(1, 48.7)	2.3	0.140	(1, 52.9)	2.00	0.165
Pop × MJ	(9, 50.1)	0.7	0.669	(9, 54.2)	1.03	0.432

Degrees of freedom (DF), F-ratio (F), and p-value (p) for each factor are shown (N = 130 plants). Significant p-values (p < 0.05) are highlighted in bold.

**Figure 4:**
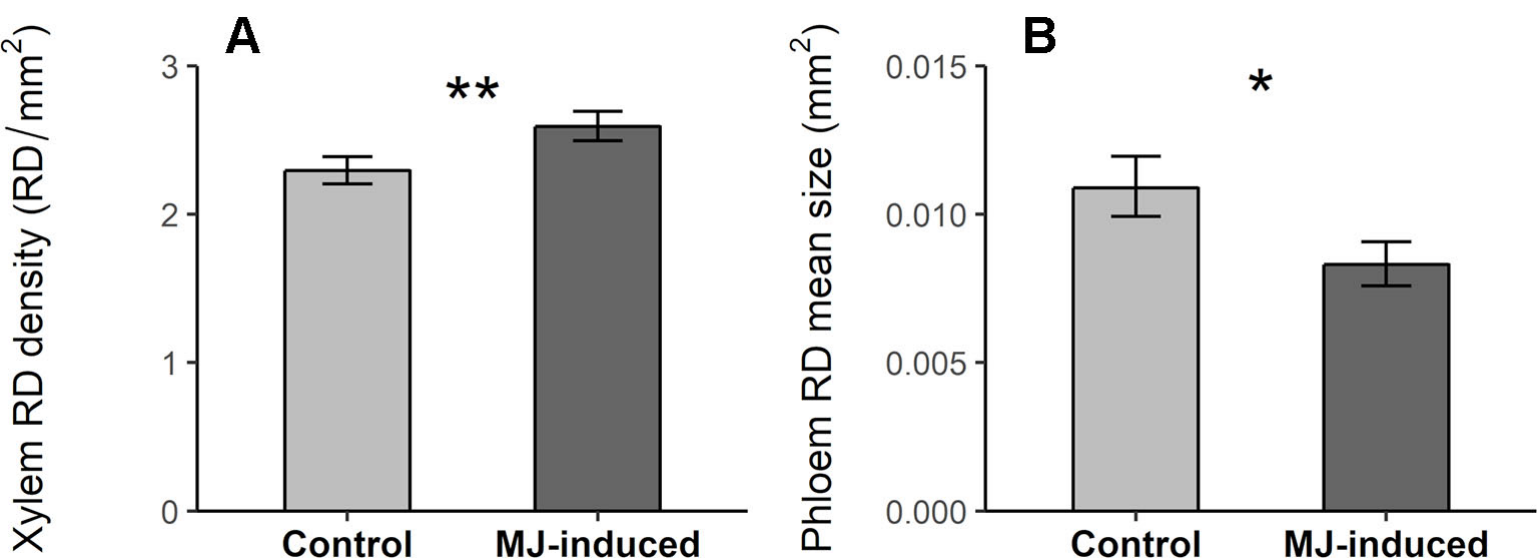
Resin duct density (RD density, RD/mm^2^) in the xylem **(A)** and resin duct mean size (RD mean size, mm^2^) in the phloem **(B)** in MJ-induced (N = 67) and control plants (N = 63) of *Pinus pinaster* from 10 populations. Bars represent least square means ± SE extracted from the mixed model. Significant p-values computed from the corresponding mixed model are indicated with asterisks (p < .01**; p < .05*).

MJ significantly increased RD density in the xylem and decreased RD mean size in the phloem (**Table 2**; **Figure 4**), but had no effect on other resin duct characteristics (**Table 2**, **Figure S1**). We did not find inter-population variation in the inducibility of resin ducts for any of the analyzed traits (non-significant Pop × MJ interaction; **Table 2**).

**Figure 5:**
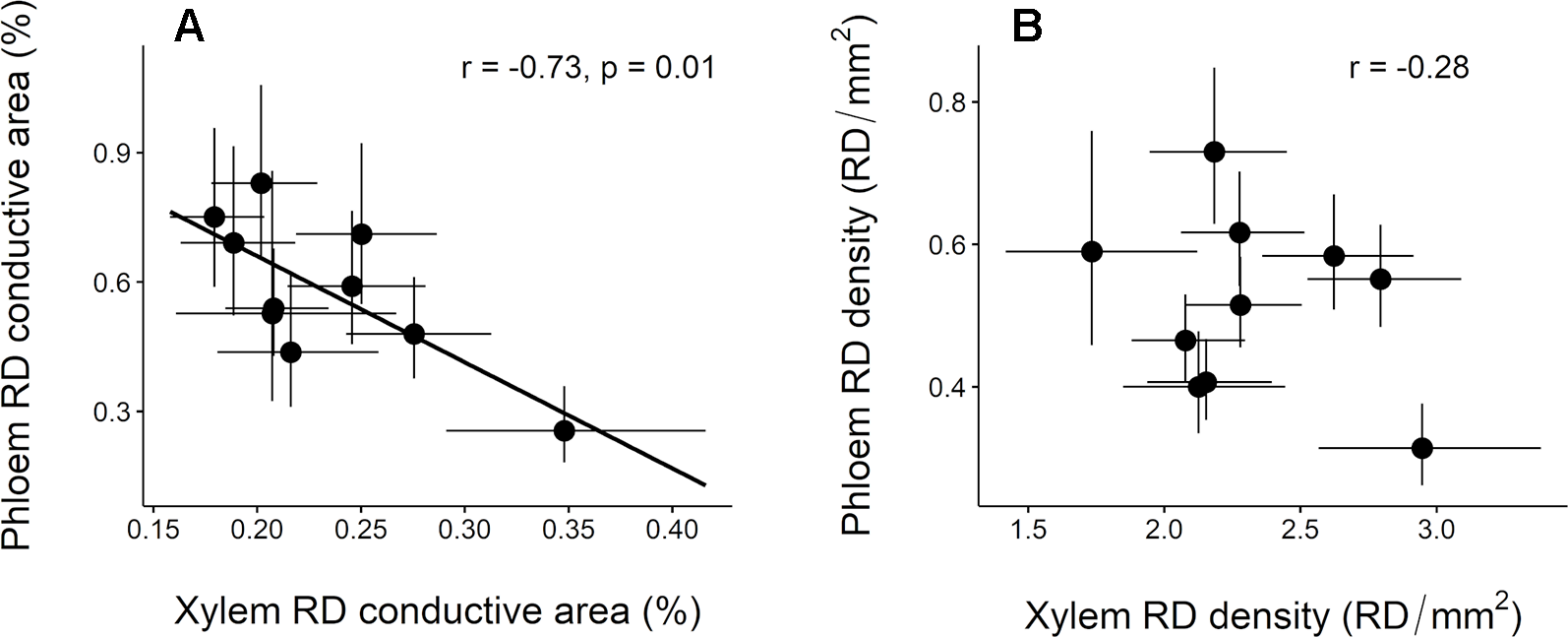
Relationship between constitutive resin duct features in the xylem and phloem across 10 *Pinus pinaster* populations. **(A)** Resin duct conductive area (RD conductive area, %) and **(B)** resin duct density (RD density, RD/mm2). Each point represents the population least square means ± SE extracted from the mixed model. Pearson correlation coefficient (r) and associated p-values (p) are shown.

Inter-population variation in constitutive RD conductive area in the phloem was significantly and negatively correlated with that in the xylem (**Figure 5A**). A similar trend was observed for RD density although the correlation was not significant (**Figure 5B**).

### Clinal Variation With Climate of Resin Duct Characteristics

**Figure 6:**
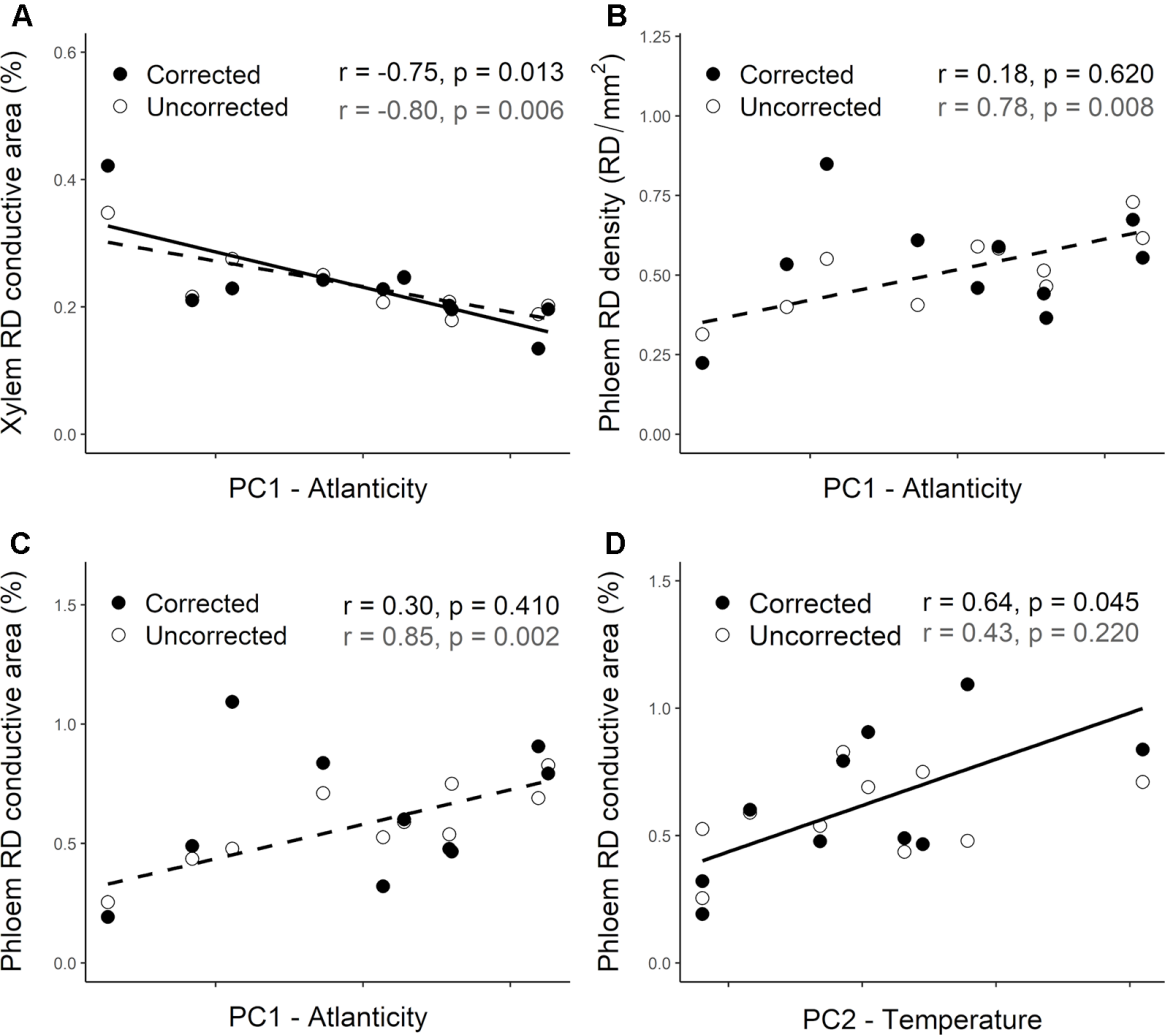
Relationship between climate indices (PC1 and PC2) and the constitutive resin duct characteristics—Resin duct density (RD density, RD/mm^2^, panel **B**) and/or conductive area (RD conductive area, %, panels **A**, **C** and **D**)—in the xylem **(A)** and phloem **(B**, **C**, **D)** across 10 *Pinus pinaster* populations. White circles represent uncorrected population least squared means, whereas dark circles represent corrected data after accounting for the population genetic structure in the corresponding mixed models. Higher values of PC1 are indicative of Atlantic climates with higher precipitation and lower thermic oscillation, whereas lower values are associated with Mediterranean climates. PC2 is positively associated with temperature and potential evapotranspiration. Pearson correlation coefficients (r) and associated p-values (p) are shown for uncorrected (gray) and corrected (black) data. Significant correlations are also represented with trend lines (dashed—uncorrected; solid—corrected).

Population differentiation in several resin duct characteristics was associated with climate across their distribution range. Before accounting for the population genetic structure, constitutive RD density and RD conductive area in the phloem were positively correlated with the PC1. However, RD conductive area in the xylem was negatively correlated with this index (**Table 3**—Uncorrected). Thus, Atlantic populations had lower RD conductive area in the xylem (**Figure 6A**—Uncorrected) and higher RD density and RD conductive area in the phloem (**Figures 6B**, **C**—Uncorrected) than Mediterranean populations. We did not find significant correlations between resin duct characteristics and the PC2.

**Table 3:** Pearson correlation coefficient (r) between constitutive resin duct characteristics—resin duct density (RD density—RD/mm^2^) and relative conductive area (RD conductive area—%)—in the xylem and phloem and climate at the origin of 10 *Pinus pinaster* populations.

	Xylem				Phloem			
	*RD density*		*RD conductive area*		*RD density*		*RD conductive area*	
	Uncorrected	Corrected	Uncorrected	Corrected	Uncorrected	Corrected	Uncorrected	Corrected
**PC1**	‑0.49	‑0.27	‑0.80**	‑0.75*	0.78**	0.18	0.85**	0.3
**PC2**	‑0.13	‑0.38	‑0.15	‑0.34	‑0.15	0.5	0.43	0.64*

Higher values of PC1 indicate higher precipitation and lower thermic oscillation (Atlantic climates), whereas lower values indicate lower precipitation and higher thermic oscillation (Mediterranean climates). PC2 is positively associated with temperature and potential evapotranspiration. Correlation coefficients between variables before (Uncorrected) and after (Corrected) accounting for the population’s genetic structure are shown. Significant Pearson correlation coefficients (r) are indicated with asterisks (p < .01**; p < .05*) and highlighted in bold font.

After accounting for the population genetic structure only RD conductive area in the xylem remained negatively correlated with the PC1 (**Table 3**; **Figure 6A**—Corrected). RD density and RD conductive area in the phloem were no longer correlated with this climate index (**Table 3**; **Figures 6B**, **C**—Corrected). Although we didn’t find any significant correlations between resin duct characteristics and the PC2 before accounting for the population structure of the species, a significant positive correlation between RD conductive area in the phloem and the PC2 emerged after including the *Q* matrix in our models (**Table 3**; **Figure 6D**—Corrected).

## Discussion

We found large inter-population variation in constitutive resin ducts, but no evidence of inter-population genetic variation in their inducibility. Of all the resin duct characteristics examined, only RD conductive area both in the xylem and in the phloem were correlated with climate at the origin of the populations after accounting for the neutral demographic history of the species. These results suggest that adaptive diversifying selection associated with heterogeneous climates may be contributing to differentiation between populations for these traits only.

### Inter-Population Variation in Resin Duct Characteristics and Their Inducibility

Inter-population variation in defensive traits is expected because allocation to defenses is costly and resource availability varies across the distribution range of the species. Thus, plants must balance resource investment between different life functions (e.g. growth, reproduction, maintenance) (Herms and Mattson, 1992; Endara and Coley, 2011). Abiotic environmental conditions may select for divergent allocation priorities and thus, heterogeneous environments would favor population differentiation in terms of defensive investment. Supporting this prediction, we found that *P. pinaster* harbors large inter-population variation in the characteristics of their resin duct system, particularly for those metrics relative to secondary growth (RD density and RD conductive area). These results also agree with previous findings. In particular, Zas et al. (2015) found that Iberian populations of this species differ greatly in xylem RD density and cortical RD mean size. Likewise, O’Neill et al. (2002) found that constitutive resin duct characteristics (area and number) had high intraspecific genetic variation in *Picea* sp. Variation among *Pinus sylvestris* L. and *P. nigra* J.F. Arnold populations in resin duct characteristics has also been reported (Martin et al., 2010; Esteban et al., 2012), although these studies did not use common gardens and thus the observed variation cannot be exclusively linked to genetic (population) effects.

The observed variation in resin ducts among *P. pinaster* populations suggests important implications in potential biotic resistance in this species. It has been suggested that resource allocation to resin ducts provides enhanced resistance to different antagonists. For instance, in North American pine species, trees that survived or avoided bark beetle attacks had more or larger resin ducts than trees that died (Kane and Kolb, 2010; Ferrenberg et al., 2014; Gaylord et al., 2015; Hood et al., 2015; Zhao and Erbilgin, 2019). Furthermore, *Picea sitchensis* provenances previously characterized as resistant to the white pine weevil (*Pissodes strobi*) exhibited a denser network of resin ducts (King et al., 2011). Enhanced resistance to other antagonistic organisms has also been associated with higher production of resin ducts (Christiansen et al., 1999; Krokene et al., 2003), but not always (Zas et al., 2015). Higher resin duct production may lead to enhanced resistance by means of increasing terpene production, one of the major chemical defensive compounds in conifers (Raffa, 2014). Indeed, we found that the density of phloem resin ducts was positively correlated with the concentration of diterpenes in the same populations (*r* = 0.69; *p-value* < 0.05; *N* = 10), which were analyzed in previous work (López-Goldar et al., 2018; López‐Goldar et al., 2019). The association between resin duct production and chemical defenses highlights the relevance of resin ducts as an important defensive trait in conifers.

The induction of resin ducts can also be crucial for conifer resistance to insects and pathogens (Christiansen et al., 1999; Krokene et al., 2003). Here we found that MJ increased RD density in the xylem, probably by means of *de novo* production of traumatic resin duct, as expected according to previous studies in *Pinus* (Vivas et al., 2012; Moreira et al., 2015b) and *Picea* species (Franceschi et al., 2002; Martin et al., 2002; Erbilgin et al., 2006; Krokene et al., 2008). In contrast, the exogenous application of MJ reduced RD mean size in the phloem compared to control untreated plants. The observed reduction in RD mean size in the phloem was surprising and may be explained as a side effect of MJ caused by enhanced resin production in the epithelial cells. It is known that MJ increases the synthesis of resin defenses (Sampedro et al., 2011; López-Goldar et al., 2018) and resin flow (Franceschi et al., 2002). Since resin is synthesized in the epithelial cells surrounding the resin duct lumen (Fahn, 1979) increased resin production and accumulation after induction could lead to the swelling of the epithelial cells reducing the lumen area of resin ducts. Further research should test this idea, exploring the effects of MJ on the activation of epithelial cells in the cortical resin ducts. Moreover, the state of hydration of resin duct epithelial cells could be affecting their lumen area. However, as previously stated in the methods section, all plants were well watered and remained in the same conditions during the experiment. Thus, this factor was unlikely contributing to the observed variation between groups.

Given the impact of induced resin defenses on resistance (Krokene et al., 2003; López-Goldar et al., 2018), the costs of their production (Sampedro et al., 2011) and the heterogeneous abiotic and biotic conditions across the distribution range of the species, variation among populations in the plastic responses to biotic stimuli was also expected. Indeed, intraspecific genetic variation among families in the inducibility of resin ducts and chemical defenses was previously reported for *P. pinaster* (Sampedro et al., 2011; Moreira et al., 2015b). In contraposition, a parallel study using the same plant material as our study (López‐Goldar et al., 2019) found scarce intraspecific genetic variation among and within populations in the inducibility of plant secondary metabolites. Similarly, we did not find a significant interaction between pine populations and the induction treatment, indicating that all populations responded similarly to MJ. Our results may suggest that stabilizing selection has been operating on the inducibility of resin ducts leading to a highly conserved response among populations.

Constitutive RD conductive area in the xylem and the phloem were negatively correlated among populations pointing to a possible trade-off between the development of resin ducts in each tissue. While resin ducts in the xylem are the main resin reservoir during mature stages, resin ducts in the primary phloem are only present during the first years of development and are an important defensive mechanism in conifer seedlings (Franceschi et al., 2005). Theory suggests that redundant and costly defensive traits may trade-off among each other, although the evidences supporting this premise are scarce (Koricheva et al., 2004; Agrawal et al., 2010). Higher allocation priorities to phloem resin ducts could therefore lead to under defend the xylem, and *vice versa*. Differences in local environmental conditions could explain divergent defensive allocation priorities in each tissue among populations. For instance, populations exposed to high biotic stress may produce more resin ducts in the primary phloem in order to ensure tree survival at early developmental stages. However, our study lacks data on biotic pressures at the origin of populations and therefore this hypothesis cannot be tested. Further studies should explore the broad existence of this pattern in this and other species and assess the possible causes underlying this trade-off.

### Adaptive Significance of Climate Gradients in Resin Duct Characteristics

Before accounting for the population genetic structure of the species, we found several significant correlations between resin duct characteristics in the phloem and xylem and the climate index PC1 (indicative of Atlantic climate). Phenotypic differences in resin duct characteristics among *P. nigra* provenances have also been associated with climate conditions in the Iberian Peninsula, with trees from colder environments producing more and larger radial resin ducts (Esteban et al., 2012). Likewise, differences in constitutive and traumatic resin duct characteristics among populations from the *Picea sitchensis* × *Picea glauca* (Moench) Voss introgression zone were found to be positively correlated with aridity and continentality at the origin of the populations (O’Neill et al., 2002).

According to the results obtained by this and other studies, it may be assumed that differential climate conditions act as a selective force contributing to the adaptive differentiation among populations (Alberto et al., 2013). However, inter-population variation does not only include adaptive but also neutral variation associated with the migratory and demographic history of the species (González‐Martínez et al., 2006; Holderegger et al., 2006). *P. pinaster*, like other European tree species, experienced severe bottlenecks during the ice ages (Gómez et al., 2005). This fact, together with its singular postglacial expansion from several relict emplacements, as well as its fragmented distribution, has led maritime pine to harbor large and geographically structured neutral genetic variation (Jaramillo-Correa et al., 2015; Serra‐Varela et al., 2015; López‐Goldar et al., 2019). As a result, the observed differentiation among populations in many phenotypic traits may not be related to enhanced tree fitness (*i.e.,* adaptation). That seems to be the case for most plant secondary metabolites in *P. pinaster*, whose genetic differentiation among populations (*Q_ST_*) was not different from that expected by genetic drift alone (*F_ST_*) (López‐Goldar et al., 2019).

When making inferences in comparative ecology, not accounting for the genetic relatedness can lead to the misinterpretation of potential clinal patterns due to the non-independence of the populations (Desdevises et al., 2003). After accounting for the population genetic structure we found that RD density in the phloem was no longer significantly correlated with the PC1 (Atlanticity). This finding indicates that the correlation between RD density in phloem and climate was probably not due to adaptive processes but rather caused by geographically structured patterns of neutral variation. Similar results were obtained for chemical defenses in this species, for which climate clines where reported to be due to neutral demographic processes in most cases (López‐Goldar et al., 2019).

The opposite situation would be those resin duct characteristics that were significantly correlated with climate even after accounting for the demographic history of the species. Such is the case of RD conductive area. In the xylem, this trait remained significantly and negatively correlated with the PC1 of Atlanticity after accounting for the neutral variation, suggesting that climate heterogeneity is contributing to inter-population differentiation of resin ducts in this tissue. In addition, a positive significant correlation between RD conductive area in the phloem and the PC2 emerged after accounting for the neutral variation. In both cases, adaptation to local climate conditions, alongside the demographic history of the species, seems to help explain the differentiation of populations in these traits. Current hypothesis on growth-defense trade-offs state that resource allocation to defenses is promoted in limiting environments to the detriment of growth capacity (Coley et al., 1985; Herms and Mattson, 1992; Endara and Coley, 2011). Our results somewhat support such a hypothesis, as populations from Atlantic regions characterized by less limiting conditions for tree growth invest less in resin duct production in the xylem than Mediterranean populations. Similarly, differentiation of populations towards higher allocation to phloem resin ducts is promoted in environments with higher temperature and potential evapotranspiration indicative of higher water stress. Moreover, we should acknowledge that resource allocation to defenses may also trade-off with investment in reproduction (Herms and Mattson, 1991; Redmond et al., 2019). Therefore, environmental conditions favoring higher reproductive investment could also limit defense production. RD conductive area is a more informative metric than RD density, as it accounts for both the density and the area of resin ducts in a tissue. Indeed, previous studies reported that a combination of smaller but denser resin ducts enhanced biotic resistance (Reed et al., 1986; Boucher et al., 2001). Accordingly, our results suggest that the relative conductive area of resin ducts has a greater adaptive and evolutionary significance than their relative number or their average size.

We must consider that separating neutral from adaptive variation in most European tree species may be challenging, as the postglacial migration history of these species parallels current environmental gradients (Serra‐Varela et al., 2015; López‐Goldar et al., 2019). Further studies in this and other conifer species should include comparative analysis of neutral *versus* quantitative variation (*F_ST_* -*Q_ST_* analysis) in resin duct characteristics to broadly understand the evolutionary origin of this anatomical trait. Moreover, assessment of adaptive genetic variation in resin ducts should also be tested in mature trees, as previous studies showed that ontogeny plays an important role modulating resin-based defenses in conifers (Erbilgin and Colgan, 2012). We should also consider that maternal effects may be contributing to the observed differences among populations. Seeds were collected on natural populations growing in highly contrasted environments, which can lead to differential transgenerational plastic effects through mechanisms of non-genetic heritance (Yakovlev et al., 2012). However, the seeds collected in each population were initially planted in a common environment from which the cuttings used here were obtained. Although, common environmental conditions have likely helped to reduce such epigenetic effects, carryover and transgenerational effects could still be occurring. Moreover, heterogeneity in the biotic environment among populations (*i.e.,* differential biotic pressure at the origin of populations) which is likely associated to climate gradients (Moreira et al., 2015a), could also contribute to explain patterns of genetic variation in this species. Unfortunately, we lack historical information on herbivory/pathogen pressures at the origin of the populations and thus this hypothesis could not be tested. We are aware that the sample size within populations in our study may limit our capacity to draw general assumptions. However, the strong relationship found between genetic differentiation in resin duct traits and climate still has great relevance in the theoretical framework of plant defense theory.

Altogether our findings show that even after accounting for the neutral variation in our models, differentiation between populations in resin duct characteristics was associated with climate gradients. Our results therefore are evidence that variation in the quantitative characteristics of this anatomical defensive trait in *P. pinaster* likely has an adaptive origin and suggests that resin duct characteristics have differentially evolved across heterogeneous climates. Although the inducibility of resin ducts is supposed to be an important mechanism conferring enhanced resistance, here we found no evidence of inter-population variation in their response to MJ. Thus, the magnitude of the biotic plasticity may not have been subject to diversifying selection, but rather widely conserved across the distribution range of *P. pinaster*. We aim to highlight the importance of accounting for the population genetic structure when inferring climate clines in phenotypic traits in species with large spatially structured neutral variation.

## Data Availability Statement

The SNP dataset analyzed in this study is publicly available in the Zenodo repository (doi: 10.5281/zenodo.1445313).

## Author Contributions

LS and RZ designed the experiment, performed the sampling, helped with the statistical analyses and the interpretation of the results, and improved the different versions of the manuscript. XL-G performed the sampling, produced the Q matrix, helped with the statistical analysis and the interpretation of the results, and contributed to improve the manuscript. CV-G processed the samples and obtained the images, measured resin duct features, performed the statistical analyses, produced the results, wrote the first draft with RZ, LS, and XL-G, and led the improvement of the manuscript along the peer-review process.

## Funding

This research was supported by the Spanish government (MINECO/FEDER) grants FUTURPIN (AGL2015-68274-C03-02-R), AdapCon (CGL2011-30182-C02-01/02) and RESILPINE (RTI2018-094691-B-C33), and by the Xunta de Galicia-GAIN grant (IN607A2016/013). CVG received financial support from the MINECO FPI Grant program (MINECO-Spain BES-2016-076624). We acknowledge support of the publication fee by the CSIC Open Access Publication Support Initiative through its Unit of Information Resources for Research (URICI).

## Conflict of Interest

The authors declare that the research was conducted in the absence of any commercial or financial relationships that could be construed as a potential conflict of interest.
