# Supplementary material for: Neutral and Climate-Driven Adaptive Processes Contribute to Explain Population Variation in Resin Duct Traits in a Mediterranean Pine Species
Source: Front Plant Sci. 2019 Dec 13;10:1613. doi: 10.3389/fpls.2019.01613 (PMC6923275; doi:10.3389/fpls.2019.01613)
Supplement: Supplementary file 1 [file Table_1.docx]

Supplementary Material

# Supplementary Tables

Table S1 Populations of *Pinus pinaster* included in the study.

| *Population* | *ID* | *Genetic Group* | *Latitude (N)* | *Longitude (W)* | *Altitude* | *P(ml)* | *T (ºC)* |
| --- | --- | --- | --- | --- | --- | --- | --- |
| Armayán | ARMY | Atlantic Spain | 43.304802 | -6.458273 | 498 | 1112 | 11.8 |
| Arenas de San Pedro | ASPE | Central Spain | 40.194822 | -5.116213 | 733 | 1318 | 14.2 |
| Cadavedo | CDVO | Atlantic Spain | 43.539965 | -6.417847 | 210 | 1316 | 13.2 |
| Coca | COCA | Central Spain | 41.254705 | -4.497827 | 800 | 454 | 12.3 |
| Mimizan | MIMI | Atlantic France | 44.134167 | -1.303167 | 37 | 1235 | 13.3 |
| Oria | ORIA | South Spain | 37.531165 | -2.351138 | 1223 | 357 | 13.1 |
| Pleucadeuc | PLEU | Atlantic France | 47.781194 | -2.343667 | 80 | 804 | 11.2 |
| Puerto de Vega | PTOV | Atlantic Spain | 43.547949 | -6.631375 | 121 | 1283 | 13.4 |
| San Cipriano de Ribaterme | SCRI | Atlantic Spain | 42.118331 | -8.36444 | 300 | 1600 | 12.3 |
| Tamrabta | TAMR | Morocco | 33.6 | -5.016667 | 1758 | 745 | 10.7 |
|  |  |  |  |  |  |  |  |

Table S2 Descriptive statistics of resin duct traits in the xylem and phloem of *Pinus pinaster* saplings both in the methyl jasmonate (MJ; N = 67) and the control (CT; N = 63) treatment. Pairs of means significantly different according to the corresponding linear mixed model are represented in bold.

|  | **RD density (RD/mm^2^)** | | **RD area (mm^2^)** | | **RD conductive area (%)** | |
| --- | --- | --- | --- | --- | --- | --- |
|  | *CT* | *MJ* | *CT* | *MJ* | *CT* | *MJ* |
| **Phloem** |  |  |  |  |  |  |
| *Mean* | 0.55 | 0.61 | **0.0139** | **0.0107** | 0.72 | 0.62 |
| *Min* | 0.23 | 0.23 | 0.0018 | 0.0027 | 0.13 | 0.09 |
| *Max* | 1.05 | 1.16 | 0.0486 | 0.0432 | 2.11 | 2.31 |
| *Std. dev* | 0.19 | 0.25 | 0.0094 | 0.0082 | 0.49 | 0.46 |
| **Xylem** |  |  |  |  |  |  |
| *Mean* | **2.43** | **2.75** | 0.0010 | 0.0010 | 0.24 | 0.26 |
| *Min* | 1.38 | 1.01 | 0.0004 | 0.0003 | 0.11 | 0.11 |
| *Max* | 4.18 | 5.38 | 0.0022 | 0.0019 | 0.51 | 0.51 |
| *Std. dev* | 0.70 | 0.95 | 0.0005 | 0.0004 | 0.10 | 0.10 |
|  |  |  |  |  |  |  |

Table S3 Pearson’s r correlations between resin duct characteristics in the phloem and xylem (N=130). Significant Pearson correlation coefficients (r) are indicated by asterisks (p < .001***; p < .01**; p < .05*).

|  | **Xylem** | | | **Phloem** | | |
| --- | --- | --- | --- | --- | --- | --- |
|  | *RD density* | *RD size* | RD conductive area | *RD density* | *RD size* | RD conductive area |
| **Xylem** |  |  |  |  |  |  |
| RD density |  |  |  |  |  |  |
| RD size | **-0.41***** |  |  |  |  |  |
| % RD area | **0.37***** | **0.65***** |  |  |  |  |
| **Phloem** |  |  |  |  |  |  |
| RD density | 0.17 | -**0.31***** | **-0.21*** |  |  |  |
| RD size | **-0.24**** | 0.14 | -0.03 | **-0.22*** |  |  |
| % RD area | -0.16 | -0.01 | -0.12 | **0.32***** | **0.79***** |  |
|  |  |  |  |  |  |  |
|  |  |  |  |  |  |  |

**Figure S1** Non-significant effects of methyl jasmonate treatment on resin duct traits. Resin duct density (RD density - RD/mm^2^) in the phloem (a), resin duct mean size (RD mean size - mm^2^) in the phloem (b) and resin duct conductive area (RD conductive area - %) in the phloem (c) and xylem (d) in MJ-induced (N = 67) and control plants (N = 63) of *Pinus pinaster* from 10 populations. Bars represent least square means ± SE extracted from the mixed model.

**
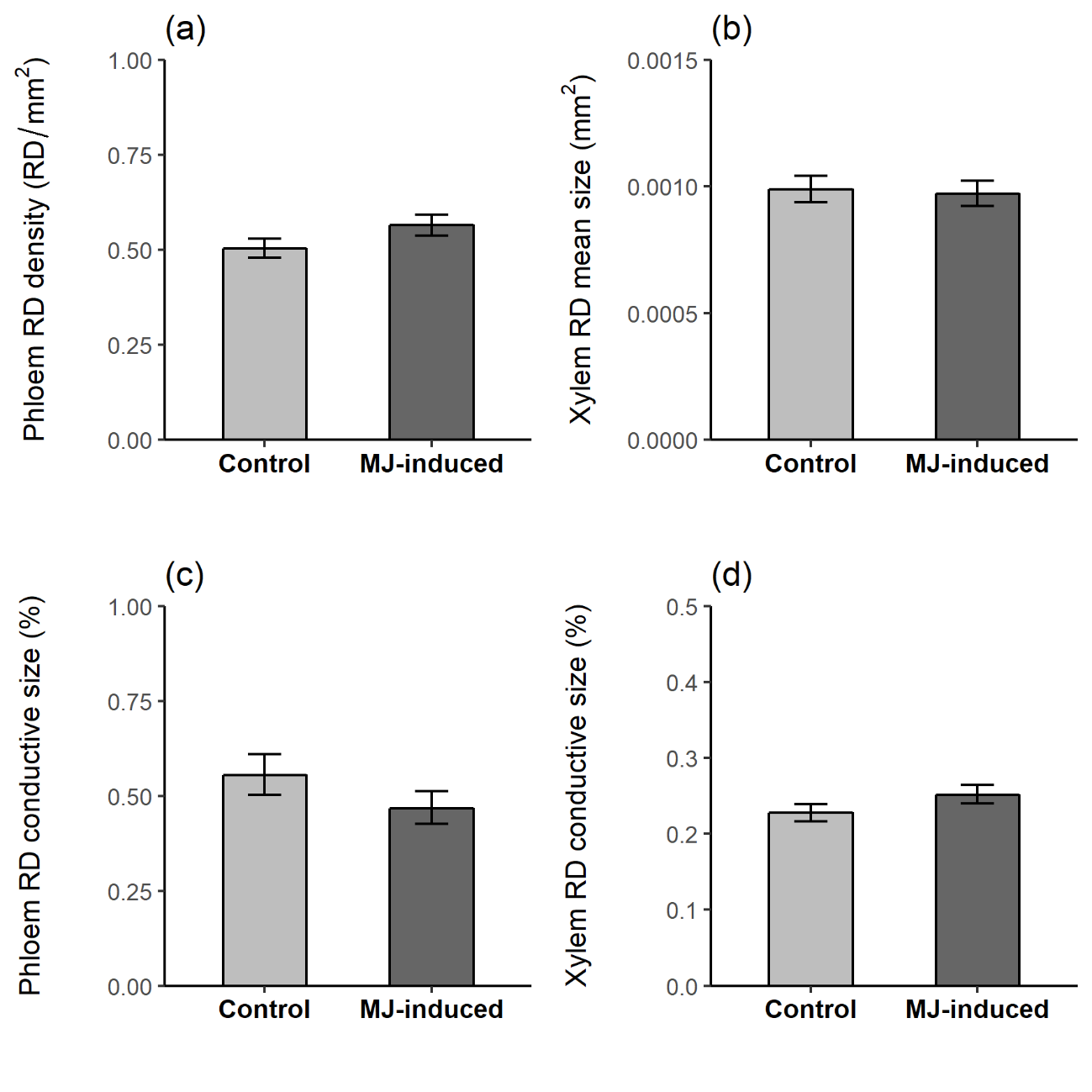
**
